# Relapses of juvenile idiopathic arthritis in adulthood: A monocentric experience

**DOI:** 10.1371/journal.pone.0298679

**Published:** 2024-05-02

**Authors:** Laura Scagnellato, Giacomo Cozzi, Ilaria Prosepe, Mariagrazia Lorenzin, Andrea Doria, Giorgia Martini, Francesco Zulian, Roberta Ramonda

**Affiliations:** 1 Rheumatology Unit, Department of Medicine DIMED, Padova University Hospital, Padova, Italy; 2 Biomedical Data Science Department, Leiden University Medical Centre, Leiden, The Netherlands; 3 Paediatric Rheumatology Unit, Department of Women’s and Children’s Health, Padova University Hospital, Padova, Italy; Siksha O Anusandhan University School of Pharmaceutical Sciences, INDIA

## Abstract

**Introduction:**

Our aim was to describe a monocentric cohort of young adult patients with juvenile idiopathic arthritis (JIA), assessing the risk of relapse after transition to adult care.

**Methods:**

We conducted a retrospective study and collected clinical, serological, and demographic data of young adult patients (18–30 years old) referred to the Transition Clinic of a single Italian centre between January 2020 and March 2023. Patients with systemic-onset JIA were excluded. Primary outcome was disease relapse, defined by Wallace criteria. Risk factors were analysed by Cox proportional hazards regression.

**Results:**

Fifty patients with age 18–30 years old were enrolled in the study and followed for a median 30 months. The median disease duration at transition was 15 years. Twenty (40%) patients were on conventional synthetic disease-modifying antirheumatic drugs (csDMARDs) and 38 (76%) were on biological DMARDs through adulthood. Twenty-three patients relapsed after transitioning to adult care for a median 9-month follow-up (IQR 0–46.5). Most relapses involved the knees (69.6%). The univariate analysis identified monoarthritis (HR 4.67, CI 1.069–20.41, p value = 0.041) as the main risk factor for relapse within the first 36 months of follow-up. Early onset, ANA positivity, past and ongoing treatment with csDMARDs or bDMARDs, therapeutic withdrawal, and disease activity within 12 months before transition did not significantly influence the risk of relapse.

**Conclusion:**

In JIA patients, the risk of relapse after transitioning to adult care remains high, irrespective of disease subtype and treatment. The main risk factor for the early occurrence of articular activity is monoarticular involvement.

## Introduction

Juvenile idiopathic arthritis (JIA) describes a diverse spectrum of rare chronic arthritides presenting in children younger than 16 years old. At present, JIA patients are classified into 7 classes according to some peculiar features both in the articular and extra-articular domains, according to the 2004 International League of Associations for Rheumatology (ILAR) classification [1, 2]. This classification system comprises systemic-onset JIA, oligoarticular JIA, polyarticular JIA, psoriatic JIA, enthesitis-related arthritis (ERA), and undifferentiated arthritis. Importantly, many paediatric patients presenting with arthritis are anti-nuclear antibody (ANA) positive and some patients with polyarticular disease are rheumatoid factor (RF) positive [3]. ANA carriers with oligoarticular involvement are at higher risk of developing chronic anterior uveitis (CUA), an asymptomatic iridocyclitis that can cause serious visual impairment and ocular complications [4]. The current ILAR classification criteria appear to be inadequate to implement JIA treatment and predict outcome [5, 6], in light of recent evidence on JIA pathogenesis. The classification and outcomes of disease phenotypes are still ill-defined in the paediatric age, and is the case in adulthood for outcomes and predictors [7, 8].

The transition from the paedriatric to the adult care is a crucial moment in the JIA disease course [9] associated with known risks of worsening global health and treatment adherence [10, 11]. Notably, Nordic Countries with a robust and free universal health care system often see high proportion of young adults previously followed in a Paediatric Clinic develop active disease and not receive appropriate rheumatological care afterwards [9].

Given the lack of specific management guidelines for adult JIA patients, adult rheumatologists usually treat JIA patients following treatment guidelines for rheumatoid arthritis (RA), albeit it is demonstrated that they are distinct clinical entities. Even though treatment guidelines do not appear to differ substantially between adult RA/psoriatic arthritis and juvenile oligoarthritis/polyarthritis, the treatment armamentarium is markedly reduced in the juvenile forms [4, 12].

Our study aimed to ascertain the risk of relapse in a cohort of young adult JIA patients who recently transitioned to adult rheumatological care.

## Materials and methods

Patients with a diagnosis of juvenile idiopathic arthritis according to the 2004 International League of Associations for Rheumatology (ILAR) criteria were recruited and classified into one of the seven classes described by the ILAR criteria [13]. The inclusion criteria were a confirmed diagnosis of JIA excluding systemic onset JIA; birthdate after 1996; on-going follow-up at our Padua University Hospital Transition Clinic. Included patients were referred to the clinic from 1^st^ January 2020 up to 30^th^ March 2023. Our transition programme consists of a discussion between the paediatric rheumatologist and the patient about transitioning to adult care upon turning 18 years old. Based on each patient’s clinical history, disease severity and compliance, the paediatric rheumatologist may suggest the patient be switched to adult care earlier, at the age of 16. If patients and their families agree, a preliminary interdisciplinary meeting is convened between the paediatric rheumatologist and an adult rheumatologist to discuss the patient’s current conditions, followed by a joint visit with the patient and their family. The patient is followed by the same adult rheumatologist for at least three years. In the transition clinic, a dedicated nurse is available to manage appointments and to help for any organisational needs.

Each patient’s clinical records were consulted in April 2023 via our online database. Records were anonymized for data analysis. Systemic onset JIA patients are referred to an adult clinic dedicated to autoinflammatory disorders and therefore they were not included in this study. The study was conducted according to the ethical standards of the ad hoc committee on human experimentation (Ethics committee for clinical experimentation at Padova University Hospital, approval number 3572/AO/15 on September 15^th^ 2015), and in compliance with the principles of the Declaration of Helsinki. All patients gave their written informed consent.

Demographic and clinical data were retrieved from electronic-based clinical charts. In detail, anti-nuclear antibody positivity, RF positivity and anti-cyclic citrullinated peptide positivity, previous joint involvement and treatments including arthrocentesis, glucocorticoids, non-steroidal anti-inflammatory drugs (NSAIDs), conventional synthetic disease modifying anti-rheumatic drugs (csDMARDs) and biologic DMARDs (bDMARDs) were recorded; csDMARDs included methotrexate, sulfasalazine, hydroxychloroquine, leflunomide, azathioprine, mesalazine; bDMARDs included all monoclonal antibodies prescribed for the treatment of JIA and its associated inflammatory manifestations, namely adalimumab, etanercept, infliximab, certolizumab, abatacept, tocilizumab, vedolizumab (for associated inflammatory bowel disease), ustekinumab (for psoriasis), secukinumab. Tapering after transition was intended as any slow decrease of csDMARDs dosage or any increased intervals between bDMARDs doses.

Clinical remission was the primary outcome and was defined according to the Wallace criteria [13]. Relapse was defined as no longer fulfilling the Wallace criteria [13] for clinical remission at one or more than one visits, with the need for a therapeutic change. For each relapse, tender joint and swollen joint counts were recorded, as well as C-reactive protein (CRP) level (mg/L). Disease activity 12 months prior to transition was evaluated retrospectively according to the same criteria.

The first disease relapse occurring during the follow-up during adult rheumatological care and subsequent treatments were recorded separately. Patients who developed arthritis requiring therapeutic intervention were compared to patients who maintained clinical remission up to the third year (36 months) of follow up in the Transition Clinic.

### Statistical analysis

Proportions were described as percentages. Continuous variables were expressed as median and interquartile range (IQR) 25–75%. Due to the low density of the dataset after the third year of follow-up, a time horizon was fixed at 36 months of follow up. Groups were compared using the Mann-Whitney test considering a significance level of 0.05. We selected a subset of clinical variables, which are known risk factors in the literature, namely sex, HLA-B27 positivity, age at diagnosis, disease duration, ANA positivity, ACPA positivity, diagnosis of oligoarticular JIA, diagnosis of polyarticular JIA, episodes of monoarthritis, diagnosis of ERA, use of NSAIDs, csDMARDs, bDMARDs, disease activity prior to transition and therapeutic reductions. Selected variables were employed to evaluate their effect on survival via Cox proportional hazards models, with a 3 years (36 months) time horizon which was chosen based on the follow-up distribution to ensure reliability. Due to the small sample size and the very low event number, all covariates were included separately in a univariate Cox proportional hazards regression model [14]. The survival analysis was conducted on R, with the survival package [15] and results are expressed in terms of hazard ratios (HR). The validity of the proportional hazards assumption was checked via the Schoenfeld residuals.

## Results

We enrolled 50 Caucasian patients followed in the Adult Transition Clinic. All patients were ≥18 years old and ≤30 years old with a median disease duration at transition of 15 years (8.75–17.25 years). Demographic and clinical characteristics are fully reported in Table 1. In detail, the cohort comprised 31 patients (62%) diagnosed with oligoarticular JIA, 14 patients (28%) with polyarticular JIA, 4 patients (8%) with enthesitis-related arthritis (ERA), and 2 (4%) with the diagnosis of psoriatic arthritis (PsA). Given the importance of the subset of patients provisionally defined in 2019 [6] as early-onset ANA positive JIA patients, we performed a subanalysis of patients who met the definition to assess their risk of relapse.

**Table 1:** Demographic and clinical characteristics of JIA patients included in the study.

	All	Early onset ANA positive	No relapse	Relapse
Total of patients, *n*	50	21	27	23
**Clinical features**				
Female sex *n (%)*	39 (78%)	19 (90.5%)	21 (77,8%)	18 (78,2%)
Age at diagnosis (years-old) *median IQR*	6 (3–12)	3 (2–4)	4 (3–12)	8 (3–11)
HLA-B27 positivity *n (%)*	5 (10%)	/	2 (7,4%)	3 (13%)
ANA positivity *n (%)*	37 (74%)	21 (100%)	20 (74%)	17 (73,9%)
Rheumatoid factor positivity *n (%)*	1 (2%)	0 (0%)	0	1 (4,3%)
ACPA positivity *n (%)*	3 (6%)	1 (4.7%)	2 (7,4%)	1 (4,3%)
Smoking status *n (%)*	3 (6%)	1 (4.7%)	2 (7,4%)	1 (4,3%)
ERA *n (%)*	4 (8%)	/	2 (7,4%)	2 (8,7%)
PsA *n (%)*	2 (4%)	/	1 (3,7%)	1 (4,3%)
Oligoarticular JIA *n (%)*	31 (62%)	10 (76.9%)	17 (63%)	14 (61%)
Polyarticular JIA *n (%)*	14 (28%)	3 (23.1%)	7 (25,9%)	7 (30,4%)
Monoarthritis *n (%)*	35 (70%)	10 (76.9%)	15 (55.5%)	20 (87,0%)
Sacroiliitis *n (%)*	1 (2%)	/	1 (4,3%)	0
Enthesitis *n (%)*	3 (6%)	/	1 (4,3%)	2 (8,7%)
Uveitis *n (%)*	16 (32%)	11 (52.3%)	9 (33,3%)	7 (30,4%)
Psoriasis *n (%)*	5 (10%)	3 (14.2%)	3 (11,1%)	2 (8,7%)
Inflammatory Bowel Disease *n (%)*	3 (6%)	1 (4.7%)	1 (4,3%)	2 (8,7%)
Disease activity 12 months before transition *n (%)*	15 (30%)	3 (14.3%)	5 (18,5%)	10 (43,4%)
Disease duration at transition mean (years), *median IQR*	15 (8.75–17.25)	17 (16–19)	16 (7–18)	13 (9–17)
**Treatment**				
Use of NSAIDs *n (%)*	36 (72%)	16 (76.2%)	15 (56,7%)	21 (91,3%)
Use of systemic glucocorticoids *n (%)*	26 (52%)	10 (47.6%)	15 (55.5%)	11 (47,8%)
Use of csDMARDs *n (%)*	46 (92%)	20 (95.2%)	26 (96,2%)	20 (86,9%)
csDMARDs exposure (years), *median IQR*	7 (4–12)	10.5 (8.5–16.25)	8 (6–12.5)	6 (2–9,5)
Arthrocentesis *n (%)*	27 (54%)	12 (57.1%)	13 (48.1%)	14 (60,8%)
bDMARD ever *n (%)*	41 (84%)	19 (90.5%)	24 (88,9%)	17 (73,9%)
Number of bDMARD lines, *median IQR*	1 (1–2)	1 (1–2)	1 (1–2)	1 (0–1)
bDMARDs exposure (years), *median IQR*	6 (1.75–10)	8 (4–13)	6 (3–10)	5 (0–9)
Ongoing csDMARD *n (%)*	21 (40%)	12 (57.1%)	11 (40,7%)	15 (65,2%)
Ongoing bDMARD *n (%)*	39 (76%)	19 (90.5%)	23 (85,1%)	15 (65,2%)
Tapering after transition *n (%)*	14 (28%)	4 (19.0%)	7 (25,9%)	7 (30,4%)

%:percentage; n: number of patients; IQR: interquartile range; smoking status: self reported daily smoking of ≥ 1 cigarettes; NA: antinuclear antibody; NSAID: non-steroidal anti-inflammatory drugs; FU: follow-up; csDMARDs: conventional synthetic disease modifying anti-rheumatic drugs; bDMARDs: biological disease modifying anti-rheumatic drugs; tapering: any slow decrease of csDMARDs dosage or any increased intervals between bDMARDs doses

Among all patients, 37 patients (74%) displayed ANA positivity; among polyarticular JIA patients, 1 patient (2%) was rheumatoid factor (RF) positive, and 3 patients (6%) were anti-cyclic citrullinated peptide antibodies (anti-CCP) positive. Some patients presented extra-articular involvement, namely uveitis (16 patients, 32%) and inflammatory bowel disease (3 patients, 6%). Around 30% of patients experienced some disease activity in the musculoskeletal domain in the 12 months preceding transition.

The great majority of patients were treated with at least one course of csDMARDs (92%) and/or bDMARDs (84%) during the disease course; twenty-seven patients (54%) underwent one or more arthrocentesis procedures. Although 76% of patients continued bDMARDS through adulthood, only 40% of patients retained the treatment with csDMARDs. Almost 30% of patients received reduced doses of csDMARDs or bDMARDs after transition.

**Fig 1:**
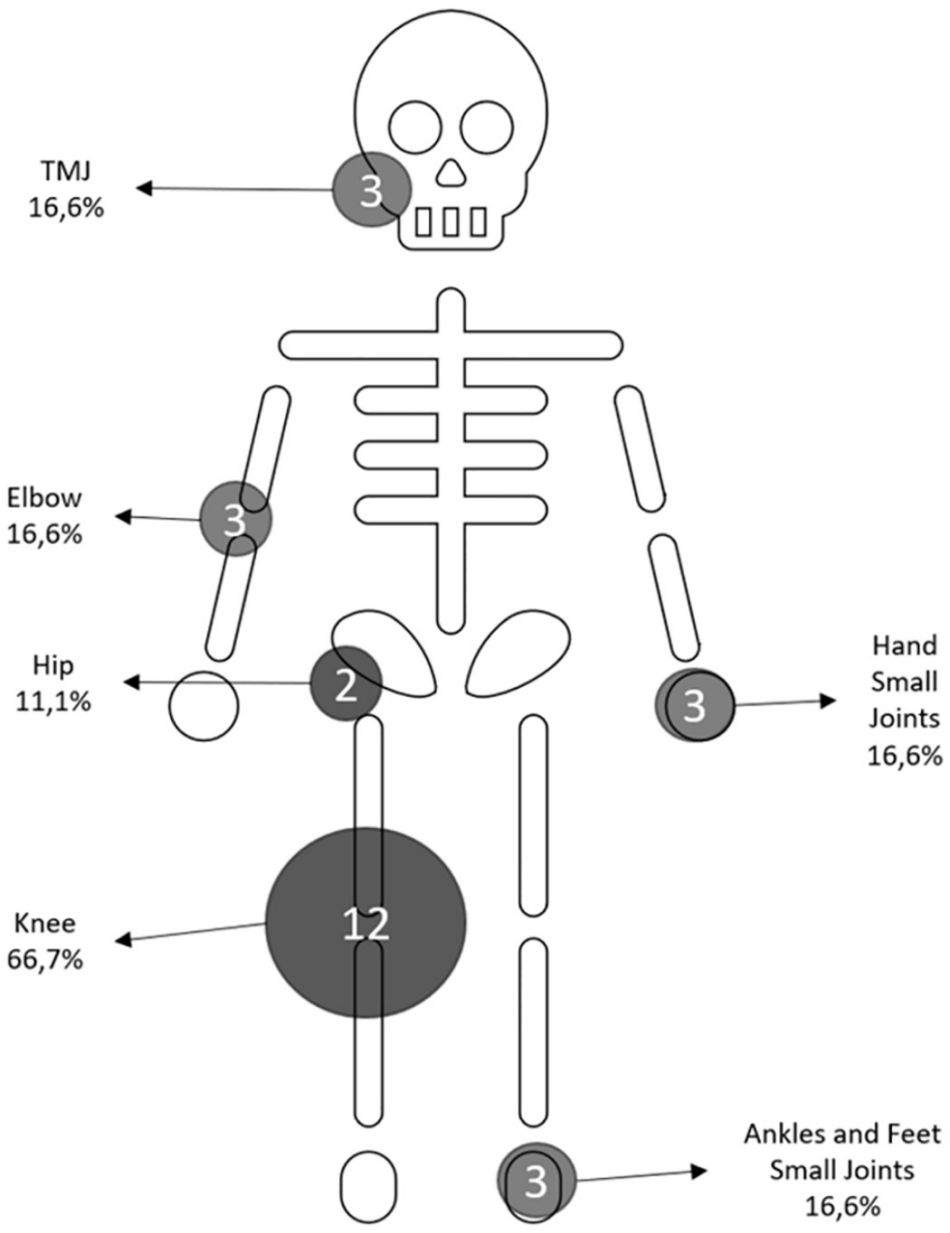
Affected joints. Joints affected by relapses in eighteen patients experiencing disease activity within 36 months after transition: the number of patients reporting pain and swelling of each joint is marked inside the circle, whose width is proportional. *TMJ*: *temporomandibular joint*. *Percentages (%) are calculated on the total of 18 patients*.

Considering the time-to-relapse survival curve, eighteen relapses occurred within the first 36 months of follow-up. The pattern of joint involvement is shown in Fig 1. Remarkably, knee involvement was predominant. At the first disease relapse after transition, the median tender joint count was 1 (IQR 1–2), as was the swollen joint count (median 1 IQR 1–1); median C-reactive protein (CRP) was 2.25 mg/L (IQR 4.75–7 mg/dL). Twenty one patients were identified as early onset ANA positive and eight (38%) relapsed within 36 months of transitioning (S1 Table).

Comparing the demographic features and clinical subtype of relapsing patients (Re) with patients in persistent remission (NRe) according to the Wallace criteria, there were not significant differences except for the history of monoarthritis, which was higher in relapsers (Re 20 patients 87% vs NRe 15 patients 55.5%, p value = 0.017). Previous therapies including systemic and local glucocorticoids, csDMARDs and bDMARDs exposure did not differ significantly between groups. Although a higher proportion of relapsers experienced some disease activity in the 12 months preceding transition, it was not statistically significant (HR 2.057, CI 0.81–5.221, p value = 0.129).

The univariable analysis (Table 2) revealed that a risk factor for relapse within the first three years after transition was monoarticular joint involvement (HR 4.67, CI 1.069–20.41, p value = 0.041). Rheumatoid factor positivity was also significantly associated with relapses but this variable could not be considered reliable because only one patient across the whole cohort displayed such a feature. At least one course of bDMARD treatment through disease history was protective for relapses (HR 0.365, CI 0.136–0.983, p-value = 0.046). Despite being slightly more frequent among relapsers (25.9% vs 30.4%), tapering was not a significant risk factor (HR = 2.709, CI 0.804–9.124, p value = 0.108).

**Table 2:** Univariable analysis on selected variables performed via Cox proportional hazards models, with a 3 years (36 months) time horizon.

Variable	Hazard Ratio	P-value	CI
Female sex	1.535	0.499	0.444–5.305
Age at diagnosis (years old)	1.024	0.672	0.923–1.132
Disease duration at transition mean (years)	0.656	0.367	0.274–1.614
Oligoarticular JIA	1.006	0.994	0.231–4.383
Polyarticular JIA	0.625	0.379	0.200–1.845
Diagnosis of Enthesitis related Arthritis	0.324	0.255	0.041–2.332
Episodes of monoarthritis	4.670	0.041	1.069–20.41
Early onset ANA positive disease	1.155	0.768	0.444–3.004
Disease activity 12 months before transition	2.057	0.129	0.81–5.221
ANA positivity	1.674	0.370	0.543–5.159
Rheumatoid factor positivity	10.08	0.034	1.193–85.19
ACPA positivity	1.28	0.811	0.169–9.718
Use of NSAIDs ever	3.469	0.097	0.797–15.10
Use of csDMARDs ever	0.529	0.318	0.151–1.848
bDMARDs ever	0.365	0.046	0.136–0.983
bDMARDs exposure (years)	0.902	0.062	0.809–1.005
Ongoing csDMARDs	1.850	0.215	0.709–4.597
Ongoing bDMARDs	0.523	0.504	0.186–1.342
Tapering after transition	2.709	0.108	0.804–9.124

JIA: juvenile idiopathic arthritis, ANA: antinuclear antibody; NSAID: non-steroidal anti-inflammatory drugs; FU: follow-up; csDMARDs: conventional synthetic disease modifying anti-rheumatic drugs; bDMARDs: biological disease modifying anti-rheumatic drugs; tapering: any decrease of csDMARDs dosage or any increased intervals between bDMARD doses

The univariate analysis performed in the early onset ANA positive group did not identify any significant risk factor for relapse (S2 Table).

## Discussion

Most patients in our cohort had oligoarticular JIA, which predominantly affects the knees and ankles, with a high rate of ANA positivity and a high risk of chronic anterior uveitis [16]. We also endeavoured to characterise the subgroup of patients meeting criteria for early-onset ANA positive JIA [6].

In the whole cohort, the median age of onset was 6 years old and 8 years old in the relapsing group, which is slightly higher than the reported mean age of onset in JIA (i.e 2–5 years old) [3]. A priori, this suggests that the early onset group might have a lower risk of relapse after transition, but this was not confirmed in the univariate analysis.

A third of young adults with JIA suffered an arthritis relapse within the first few years after transitioning to adult care, despite most of the cohort being treated with DMARDs both in childhood and adulthood. This is consistent with previous findings of similar rates of active disease in adulthood [9, 17, 18]. Although the outcomes were not influenced by disease subtype, age of onset, ANA, rheumatoid factor positivity or HLB27 positivity, they were by the occurrence of monoarthritis across disease subtypes. These results partially contradict with previous reports suggesting that clinical remission is associated with oligoarticular persistent disease [18, 19] and that HLAB27 and RF positivity correlates with increased disease activity and damage [19–21]. However, they seem consistent with the scoping review of Gieling et al. showing no correlation between disease subtype and risk of relapse after treatment retrieval in childhood [22]. Unfortunately, the early-onset ANA positivity did not correlate with the risk of relapse in our cohort. Moreover, in this subgroup, neither monoarthritis was a significant risk factor for relapse and no other clinical factors predicted a higher risk of joint activity after transition.

We hypothesised that the reason why monoarticular disease relapses are more frequent might be that it is treated less aggressively and that treatment is discontinued earlier. These patients may have received more infiltrations and less systemic treatment, resulting in localised damage, even though our data do not support this hypothesis. Regardless of treatment, after twenty years of loading and subclinical inflammation, these joints are particularly prone to develop arthritis after minimal *stimuli*, as if the local synovial response to microdamage had been skewed from a normal repair process to an abnormal response. We hypothesised that this susceptibility may be partly genetic and partly morphologic; molecular studies may provide a better understanding. We endeavour to further investigate these joints in future studies using advanced imaging and molecular biology techniques to test this hypothesis.

Our patients were treated with bDMARDs for a median of 6 years, whereas disease duration was much longer, suggesting that bDMARDs were introduced some years after disease onset. A previous study showed that patients who received early bDMARDs treatment, regardless of disease subtype, had better outcome in terms of clinical remission and damage [19]. Thus, we hypothesised that bDMARDs ought to be introduced early in childhood even in JIA patients with monoarticular involvement.

Patients currently transitioning to adult care are those with more aggressive disease, who require ongoing follow-up, whereas patients with milder disease phenotypes are successfully treated in childhood and are lost to follow-up [8, 19]. The immediate implication is that treatment tapering should be considered cautiously in the subset of JIA patients requiring adult follow-up, particularly those with predominant monoarticular involvement. Moreover, this reinforces the idea that existing treatment guidelines for paediatric JIA may not be suited for adult patients, as predictors of relapses might not be the same [4, 12, 23]. Relapses in our cohort did not appear to be linked to treatment tapering or interruption, a leading cause of disease recurrence [24]. Nevertheless, our study was not designed to detect treatment adherence, and the lack of compliance cannot be excluded in these young patients [25].

Some conflicting findings in childhood and adulthood suggest that the immune dysfunction driving the disease in adult JIA may be distinct from that observed in their younger counterparts and other adult articular diseases. Indeed, transitioning patients experience long and recurrent episodes of joint inflammation, and are exposed to immunomodulatory agents during a phase of growth and development. Therefore, management guidelines for JIA, RA, PsA and ankylosing spondylitis are fundamentally inadequate.

Our study has several limitations, and namely, its retrospective design, small sample size, and sample heterogeneity. Finally, our evaluation did not include patient-reported outcomes, treatment adherence/compliance, and validated imaging assessments of joint damage.

In conclusion, our analysis suggests that non systemic onset JIA patients still have a high risk of disease relapse in adulthood and treatment should be tapered cautiously, particularly in case of monoarticular preponderant disease. Larger prospective are needed to confirm our findings.

## Supporting information

S1 Table Demographic and clinical information on early onset ANA positive JIA patients. (DOCX)

S2 Table Univariate analysis on selected variables performed via Cox proportional hazards models, with a 3 years (36 months) time horizon. (DOCX)

S1 Data (ODT)

S1 File (PDF)

S1 Checklist STROBE statement—Checklist of items that should be included in reports of *cohort studies*. (DOC)
