# Supplementary material for: Relapses of juvenile idiopathic arthritis in adulthood: A monocentric experience
Source: PLoS One. 2024 May 2;19(5):e0298679. doi: 10.1371/journal.pone.0298679 (PMC11065285; doi:10.1371/journal.pone.0298679)
Supplement: S1 Table — (DOCX) [file pone.0298679.s001.docx]

S1 Table. Demographic and clinical information on early onset ANA positive JIA patients

| Variable | Total (21) | No relapse (13) | Relapse (8) |
| --- | --- | --- | --- |
| Female sex *n (%)* | 19 (90.5%) | 11 (84.6%) | 8 (100%) |
| Age at diagnosis  (years-old) *median IQR* | 3 (2-4) | 3 (23.1%) | 2 (25%) |
| Rheumatoid factor positivity *n (%)* | 0 (0%) | 0 | 0 |
| ACPA positivity *n (%)* | 1 (4.7%) | 0 | 1 (12.5%) |
| Chronic smoking *n (%)* | 1 (4.7%) | 1 (8.0%) | 0 |
| Oligoarticular JIA *n (%)* | 15 (71.4%) | 10 (76.9%) | 5 (62.5%) |
| Polyarticular JIA *n (%)* | 6 (28.6%) | 3 (23.1%) | 3 (37.5%) |
| Monoarthritis *n (%)* | 17 (80.9%) | 10 (76.9%) | 7 (87.5%) |
| Uveitis *n (%)* | 11 (52.3%) | 7 (53.8%) | 4 (50%) |
| Psoriasis *n (%)* | 3 (14.2%) | 3 (23.1%) | 0 |
| Inflammatory Bowel Disease *n (%)* | 1 (4.7%) | 0 | 1 (12.5%) |
| Disease activity 12 months before transition *n (%)* | 3 (14.3%) | 1 (8.0%) | 2 (25%) |
| Disease duration at transition mean (years), *median IQR* | 17 (16-19) | 17 (16-19) | 17 (16-19) |
| **Treatment** | | | |
| Use of csDMARDs *n (%)* | 20 (95.2%) | 12 (92.3%) | 8 (100%) |
| csDMARDs exposure (years), *median IQR* | 10.5 (8.5-16.25) | 11 (9.5-18) | 9.5 (6-18.2) |
| Arthrocentesis *n (%)* | 12 (57.1%) | 7 (53.8%) | 5 (62.5%) |
| bDMARD ever *n (%)* | 19 (90.5%) | 13 (100%) | 6 (75%) |
| bDMARDs exposure (years), *median IQR* | 8 (4-13) | 10 (5-13) | 3.5 (2-9) |
| Ongoing csDMARD *n (%)* | 12 (57.1%) | 7 (53.8%) | 5 (62.5%) |
| Ongoing bDMARD *n (%)* | 19 (90.5%) | 13 (100%) | 6 (75%) |
| Tapering after transition *n (%)* | 4 (19.0%) | 2 (15.1%) | 2 (25%) |

*%:percentage; n: number of patients; IQR: interquartile range; chronic smoking: self reported daily smoking of one or more cigarettes; NA: antinuclear antibody; NSAID: non-steroidal anti-inflammatory drugs; FU: follow-up; csDMARDs: conventional synthetic disease modifying anti-rheumatic drugs including methotrexate, sulfasalazine, cyclosporine, azathioprine, hydroxychloroquine; bDMARDs: biological disease modifying anti-rheumatic drugs including adalimumab, infliximab, etanercept, certolizumab, abatacept, tocilizumab, vedolizumab and ustekinumab; tapering: any tapering of csDMARDs or any increased intervals between bDMARD doses*
