# Supplementary material for: Relapses of juvenile idiopathic arthritis in adulthood: A monocentric experience
Source: PLoS One. 2024 May 2;19(5):e0298679. doi: 10.1371/journal.pone.0298679 (PMC11065285; doi:10.1371/journal.pone.0298679)
Supplement: S2 Table — (DOCX) [file pone.0298679.s002.docx]

S2 Table. Univariate analysis on selected variables performed via Cox proportional hazards models, with a 3 years (36 months) time horizon

| Variable | Hazard Ratio | P-value | CI |
| --- | --- | --- | --- |
| Polyarticular JIA | 0.825 | 0.819 | 0.159 -4.272 |
| Episodes of monoarthritis | 2.165 | 0.478 | 0.256-18.33 |
| Disease activity 12 months before transition | 1.784 | 0.492 | 0.342-9.297 |
| Use of csDMARDs ever | 0 | 0.999 | / |
| bDMARDs ever | 0 | 0.999 | / |
| Ongoing csDMARDs | 2.836 | 0.218 | 0.54 14.9 |
| Ongoing bDMARDs | 0 | 0.999 | / |
| Tapering after transition | 4.524 | 0.105 | 0.728 28.12 |

*JIA: juvenile idiopathic arthritis, ANA: antinuclear antibody; NSAID: non-steroidal anti-inflammatory drugs; FU: follow-up; csDMARDs: conventional synthetic disease modifying anti-rheumatic drugs including methotrexate, sulfasalazine, cyclosporine, azathioprine, hydroxychloroquine; bDMARDs: biological disease modifying anti-rheumatic drugs including adalimumab, infliximab, etanercept, certolizumab, abatacept, tocilizumab, vedolizumab and ustekinumab; tapering: any tapering of csDMARDs or any increased intervals between bDMARD doses*
