# Supplementary material for: Relapses of juvenile idiopathic arthritis in adulthood: A monocentric experience
Source: PLoS One. 2024 May 2;19(5):e0298679. doi: 10.1371/journal.pone.0298679 (PMC11065285; doi:10.1371/journal.pone.0298679)
Supplement: S1 File — (PDF) [file pone.0298679.s004.pdf]

REGIONE DEL VENETO-AZIENDA OSPEDALIERA DI PADOVA
COMITATO ETICO PER LA SPERIMENTAZIONE CLINICA DELLA PROVINCIA DI PADOVA

Spett.le CD Pharma Group Srl
Piazza De Angeli, 7
20146 MILANO

Chiarissimo
Prof. Leonardo Punzi
Divisione di Reumatologia
Sede

Oggetto: comunicazione parere sullo studio clinico: "Progetto GISEA – Registro online per lo studio dei pazienti affetti da artropatia infiammatoria in trattamento con farmaci biologici e/o DMARDs tradizionali". (3572/AO/15)

Con la presente si invia, in allegato, il parere che il Comitato Etico per la Sperimentazione clinica della Provincia di Padova ha espresso nella seduta del **10 Settembre 2015**

Nel caso di parere favorevole, si rammenta che per gli studi clinici sponsorizzati da Case Farmaceutiche oppure nel caso di Associazioni, Fondazioni, Enti Pubblici e Privati è possibile procedere alla loro esecuzione soltanto dopo stipula di convenzione con l'Azienda Ospedaliera di Padova che in forza della Deliberazione del Direttore Generale n.203 del 20.03.02 vale quale autorizzazione allo svolgimento presso l'Azienda Ospedaliera di Padova dell'attività di sperimentazione o di studio osservazionale.

Per l'espletamento dell'aspetto amministrativo inerente la convenzione si prega di contattare il Nucleo per la Ricerca Clinica di competenza.

Segreteria Scientifica
del Comitato etico per la Sperimentazione Clinica
della Provincia di Padova
(Dott.ssa Lodovica Gambato)

REGIONE DEL VENETO
Azienda Ospedaliera di Padova
Comitato Etico per la Sperimentazione
SEGRETERIA

Via Giustiniani, 1 – 35128 Padova Tel. 0498212341-42 fax 0498212827
e-mail: ce.sperimentazione@sanita.padova.it

REGIONE DEL VENETO-AZIENDA OSPEDALIERA DI PADOVA
COMITATO ETICO PER LA SPERIMENTAZIONE CLINICA DELLA PROVINCIA DI PADOVA

CRO: CD Pharma Group Srl

Importo rimborso spese: non sono previste prestazioni extraroutinarie, trattasi di registro osservazionale.

Il Comitato, presa visione del protocollo e della documentazione allegata, esprime da un punto di vista etico-scientifico **parere favorevole** sullo studio clinico presentato.
Nell'Informativa e manifestazione del consenso al trattamento dei dati personali e sensibili si chiede di togliere "paziente N. ___________", in considerazione del fatto che riportare l'abbinamento tra nominativo e codice identificativo in un documento che va in cartella, e non solo, può costituire una fonte che lede il diritto alla riservatezza.
Si raccomanda, inoltre, di inserire i riferimenti locali prima della somministrazione dei fogli informativi/moduli di consenso informato.
Si rileva, infine, che non sono stati inviati il Curriculum vitae e la Dichiarazione sul conflitto d'interessi del responsabile locale dello studio, che dovranno essere forniti.

Si ricorda che:
- Si ritiene indispensabile che le informazioni al paziente vengano analiticamente illustrate dal medico e discusse più ampiamente possibile.
- Il paziente relativamente al consenso informato, è libero di consultarsi con persona/e di sua fiducia.
- Il Ricercatore è tenuto a comunicare al Comitato l'inizio, la fine dello studio nonché a fornire un rapporto annuale ed un consuntivo finale scritti. Si rammenta inoltre che rientra tra le disposizioni di legge la segnalazione di tutte le reazioni avverse in corso di studio.
- La validità dell'autorizzazione è subordinata alle disposizioni contenute nella legislazione vigente.
- Tutti gli studi devono avere l'adeguata copertura finanziaria.

Segreteria Scientifica del Comitato Etico
per la Sperimentazione Clinica
(Dr.ssa Lodovica Gambato)

Lodovica Gambato

Il Presidente del Comitato Etico
per la Sperimentazione Clinica
(Dr.ssa Anna Chiara Frigo)

Anna Chiara Frigo

Copia conforme originale
