# Supplementary material for: Relapses of juvenile idiopathic arthritis in adulthood: A monocentric experience
Source: PLoS One. 2024 May 2;19(5):e0298679. doi: 10.1371/journal.pone.0298679 (PMC11065285; doi:10.1371/journal.pone.0298679)
Supplement: Supplementary file 6 [file pone.0298679.s003.odt]

Table 1. Demographic and clinical characteristics of JIA patients included in the study 

|  | All | Early onset ANA positive | No relapse | Relapse |
| --- | --- | --- | --- | --- |
| Total of patients, n | 50 | 21 | 27 | 23 |
| Clinical features |
| Female sex n (%) | 39 (78%) | 19 (90.5%) | 21 (77,8%) | 18 (78,2%) |
| Age at diagnosis (years-old) median IQR | 6 (3-12) | 3 (2-4) | 4 (3-12) | 8 (3-11) |
| HLA-B27 positivity n (%) | 5 (10%) | / | 2 (7,4%) | 3 (13%) |
| ANA positivity n (%) | 37 (74%) | 21 (100%) | 20 (74%) | 17 (73,9%) |
| Rheumatoid factor positivity n (%) | 1 (2%) | 0 (0%) | 0 | 1 (4,3%) |
| ACPA positivity n (%) | 3 (6%) | 1 (4.7%) | 2 (7,4%) | 1 (4,3%) |
| Smoking status n (%) | 3 (6%) | 1 (4.7%) | 2 (7,4%) | 1 (4,3%) |
| ERA n (%) | 4 (8%) | / | 2 (7,4%) | 2 (8,7%) |
| PsA n (%) | 2 (4%) | / | 1 (3,7%) | 1 (4,3%) |
| Oligoarticular JIA n (%) | 31 (62%) | 10 (76.9%) | 17 (63%) | 14 (61%) |
| Polyarticular JIA n (%) | 14 (28%) | 3 (23.1%) | 7 (25,9%) | 7 (30,4%) |
| Monoarthritis n (%) | 35 (70%) | 10 (76.9%) | 15 (55.5%) | 20 (87,0%) |
| Sacroiliitis n (%) | 1 (2%) | / | 1 (4,3%) | 0 |
| Enthesitis n (%) | 3 (6%) | / | 1 (4,3%) | 2 (8,7%) |
| Uveitis n (%) | 16 (32%) | 11 (52.3%) | 9 (33,3%) | 7 (30,4%) |
| Psoriasis n (%) | 5 (10%) | 3 (14.2%) | 3 (11,1%) | 2 (8,7%) |
| Inflammatory Bowel Disease n (%) | 3 (6%) | 1 (4.7%) | 1 (4,3%) | 2 (8,7%) |
| Disease activity 12 months before transition n (%) | 15 (30%) | 3 (14.3%) | 5 (18,5%) | 10 (43,4%) |
| Disease duration at transition mean (years), median IQR | 15 (8.75-17.25) | 17 (16-19) | 16 (7-18) | 13 (9-17) |
| Treatment |
| Use of NSAIDs n (%) | 36 (72%) | 16 (76.2%) | 15 (56,7%) | 21 (91,3%) |
| Use of systemic glucocorticoids n (%) | 26 (52%) | 10 (47.6%) | 15 (55.5%) | 11 (47,8%) |
| Use of csDMARDs n (%) | 46 (92%) | 20 (95.2%) | 26 (96,2%) | 20 (86,9%) |
| csDMARDs exposure (years), median IQR | 7 (4-12) | 10.5 (8.5-16.25) | 8 (6-12.5) | 6 (2-9,5) |
| Arthrocentesis n (%) | 27 (54%) | 12 (57.1%) | 13 (48.1%) | 14 (60,8%) |
| bDMARD ever n (%) | 41 (84%) | 19 (90.5%) | 24 (88,9%) | 17 (73,9%) |
| Number of bDMARD lines, median IQR | 1 (1-2) | 1 (1-2) | 1 (1-2) | 1 (0-1) |
| bDMARDs exposure (years), median IQR | 6 (1.75-10) | 8 (4-13) | 6 (3-10) | 5 (0-9) |
| Ongoing csDMARD n (%) | 21 (40%) | 12 (57.1%) | 11 (40,7%) | 15 (65,2%) |
| Ongoing bDMARD n (%) | 39 (76%) | 19 (90.5%) | 23 (85,1%) | 15 (65,2%) |
| Tapering after transition n (%) | 14 (28%) | 4 (19.0%) | 7 (25,9%) | 7 (30,4%) |

%:percentage; n: number of patients; IQR: interquartile range; smoking status: self reported daily smoking of  ≥ 1 cigarettes; NA: antinuclear antibody; NSAID: non-steroidal anti-inflammatory drugs; FU: follow-up; csDMARDs: conventional synthetic disease modifying anti-rheumatic drugs; bDMARDs: biological disease modifying anti-rheumatic drugs; tapering: any slow decrease of csDMARDs dosage or any increased intervals between bDMARDs doses

Table 2. Univariable analysis on selected variables performed via Cox proportional hazards models, with a 3 years (36 months) time horizon

| Variable | Hazard Ratio | P-value | CI |
| --- | --- | --- | --- |
| Female sex | 1.535 | 0.499 | 0.444-5.305 |
| Age at diagnosis (years old) | 1.024 | 0.672 | 0.923-1.132 |
| Disease duration at transition mean (years) | 0.656 | 0.367 | 0.274-1.614 |
| Oligoarticular JIA | 1.006 | 0.994 | 0.231-4.383 |
| Polyarticular JIA | 0.625 | 0.379 | 0.200-1.845 |
| Diagnosis of Enthesitis related Arthritis | 0.324 | 0.255 | 0.041-2.332 |
| Episodes of monoarthritis | 4.670 | 0.041 | 1.069-20.41 |
| Early onset ANA positive disease | 1.155 | 0.768 | 0.444-3.004 |
| Disease activity 12 months before transition | 2.057 | 0.129 | 0.81-5.221 |
| ANA positivity | 1.674 | 0.370 | 0.543-5.159 |
| Rheumatoid factor positivity | 10.08 | 0.034 | 1.193-85.19 |
| ACPA positivity | 1.28 | 0.811 | 0.169-9.718 |
| Use of NSAIDs ever | 3.469 | 0.097 | 0.797-15.10 |
| Use of csDMARDs ever | 0.529 | 0.318 | 0.151-1.848 |
| bDMARDs ever | 0.365 | 0.046 | 0.136-0.983 |
| bDMARDs exposure (years) | 0.902 | 0.062 | 0.809-1.005 |
| Ongoing csDMARDs | 1.850 | 0.215 | 0.709-4.597 |
| Ongoing bDMARDs | 0.523 | 0.504 | 0.186-1.342 |
| Tapering after transition | 2.709 | 0.108 | 0.804-9.124 |

JIA: juvenile idiopathic arthritis, ANA: antinuclear antibody; NSAID: non-steroidal anti-inflammatory drugs; FU: follow-up; csDMARDs: conventional synthetic disease modifying anti-rheumatic drugs; bDMARDs: biological disease modifying anti-rheumatic drugs; tapering: any decrease of csDMARDs dosage or any increased intervals between bDMARD doses

S1 Table. Demographic and clinical information on early onset ANA positive JIA patients 

| Variable | Total (21) | No relapse (13) | Relapse (8) |
| --- | --- | --- | --- |
| Female sex n (%) | 19 (90.5%) | 11 (84.6%) | 8 (100%) |
| Age at diagnosis (years-old) median IQR | 3 (2-4) | 3 (23.1%) | 2 (25%) |
| Rheumatoid factor positivity n (%) | 0 (0%) | 0 | 0 |
| ACPA positivity n (%) | 1 (4.7%) | 0 | 1 (12.5%) |
| Chronic smoking n (%) | 1 (4.7%) | 1 (8.0%) | 0 |
| Oligoarticular JIA n (%) | 15 (71.4%) | 10 (76.9%) | 5 (62.5%) |
| Polyarticular JIA n (%) | 6 (28.6%) | 3 (23.1%) | 3 (37.5%) |
| Monoarthritis n (%) | 17 (80.9%) | 10 (76.9%) | 7 (87.5%) |
| Uveitis n (%) | 11 (52.3%) | 7 (53.8%) | 4 (50%) |
| Psoriasis n (%) | 3 (14.2%) | 3 (23.1%) | 0 |
| Inflammatory Bowel Disease n (%) | 1 (4.7%) | 0 | 1 (12.5%) |
| Disease activity 12 months before transition n (%) | 3 (14.3%) | 1 (8.0%) | 2 (25%) |
| Disease duration at transition mean (years), median IQR | 17 (16-19) | 17 (16-19) | 17 (16-19) |
| Treatment |
| Use of csDMARDs n (%) | 20 (95.2%) | 12 (92.3%) | 8 (100%) |
| csDMARDs exposure (years), median IQR | 10.5 (8.5-16.25) | 11 (9.5-18) | 9.5 (6-18.2) |
| Arthrocentesis n (%) | 12 (57.1%) | 7 (53.8%) | 5 (62.5%) |
| bDMARD ever n (%) | 19 (90.5%) | 13 (100%) | 6 (75%) |
| bDMARDs exposure (years), median IQR | 8 (4-13) | 10 (5-13) | 3.5 (2-9) |
| Ongoing csDMARD n (%) | 12 (57.1%) | 7 (53.8%) | 5 (62.5%) |
| Ongoing bDMARD n (%) | 19 (90.5%) | 13 (100%) | 6 (75%) |
| Tapering after transition n (%) | 4 (19.0%) | 2 (15.1%) | 2 (25%) |

%:percentage; n: number of patients; IQR: interquartile range; chronic smoking: self reported daily smoking of one or more cigarettes; NA: antinuclear antibody; NSAID: non-steroidal anti-inflammatory drugs; FU: follow-up; csDMARDs: conventional synthetic disease modifying anti-rheumatic drugs including methotrexate, sulfasalazine, cyclosporine, azathioprine, hydroxychloroquine; bDMARDs: biological disease modifying anti-rheumatic drugs including adalimumab, infliximab, etanercept, certolizumab, abatacept, tocilizumab, vedolizumab and ustekinumab; tapering: any tapering of csDMARDs or any increased intervals between bDMARD doses

S2 Table. Univariate analysis on selected variables performed via Cox proportional hazards models, with a 3 years (36 months) time horizon

| Variable | Hazard Ratio | P-value | CI |
| --- | --- | --- | --- |
| Polyarticular JIA | 0.825 | 0.819 | 0.159 -4.272 |
| Episodes of monoarthritis | 2.165 | 0.478 | 0.256-18.33 |
| Disease activity 12 months before transition | 1.784 | 0.492 | 0.342-9.297 |
| Use of csDMARDs ever | 0 | 0.999 | / |
| bDMARDs ever | 0 | 0.999 | / |
| Ongoing csDMARDs | 2.836 | 0.218 | 0.54       14.9 |
| Ongoing bDMARDs | 0 | 0.999 | / |
| Tapering after transition | 4.524 | 0.105 | 0.728      28.12 |

JIA: juvenile idiopathic arthritis, ANA: antinuclear antibody; NSAID: non-steroidal anti-inflammatory drugs; FU: follow-up; csDMARDs: conventional synthetic disease modifying anti-rheumatic drugs including methotrexate, sulfasalazine, cyclosporine, azathioprine, hydroxychloroquine; bDMARDs: biological disease modifying anti-rheumatic drugs including adalimumab, infliximab, etanercept, certolizumab, abatacept, tocilizumab, vedolizumab and ustekinumab; tapering: any tapering of csDMARDs or any increased intervals between bDMARD doses

Fig 1. Affected joints. Joints affected by relapses in eighteen patients experiencing disease activity within 36 months after transition: the number of patients reporting pain and swelling of each joint is marked inside the circle, whose width is proportional. TMJ: temporomandibular joint. Percentages (%) are calculated on the total of 18 patients.  At the first disease relapse after transition, the median tender joint count was 1 (IQR 1-2), as was the swollen joint count (median 1 IQR 1-1); median C-reactive protein (CRP) was 2.25 mg/L (IQR 4.75-7 mg/dL). Twenty one patients were identified as early onset ANA positive and eight (38%) relapsed within 36 months of transitioning
